# The Health Behaviours of Students from Selected Countries—A Comparative Study

**DOI:** 10.3390/nursrep11020039

**Published:** 2021-05-31

**Authors:** Małgorzata Lesińska-Sawicka, Ewa Pisarek, Małgorzata Nagórska

**Affiliations:** 1 Department of Nursing, State University of Applied Sciences in Piła, 64-920 Piła, Poland; mlesinska@puss.pila.pl (M.L.-S.); ewapisarek74@wp.pl (E.P.); 2 Institute of Medical Sciences, Medical College of Rzeszow University, 35-959 Rzeszow, Poland

**Keywords:** health, lifestyle, students, health behaviour

## Abstract

Health behaviour defined as any behaviour that may affect an individual’s physical and mental health or any behaviour that an individual believes may affect their physical health. It is strongly related to their culture and plays a major role in shaping all health and illness-related behaviour. The purpose of the study was to compare and evaluate the lifestyles of students from multiple countries. The proposed work will determine the deficits in health behaviors undertaken by students. The survey was carried out from December 2016 to March 2017 comprising 532 students from Poland, Hungary, Turkey, and Greece. The sample was selected using the snowball method: a link to the online questionnaire was sent to students from the given countries via the Internet. For some participants, who did not have access to the online questionnaire, printed copies were used instead. As a method was used a diagnostic survey and the survey technique. The opinions of students were measured using the 5-level Likert scale with a neutral option. Students undertook health-promoting activities, but also list behaviours that did not contribute to strengthening their health. Students were shown to have the greatest problems with physical health behaviours and health prevention. There were noticeable differences in the lifestyle of students from different countries.

## 1. Introduction

Health behaviours can be defined as the reactive, habitual, or intentional forms of human activity, which remain significantly intertwined with health. Health behaviours include both pro-health activities (e.g., healthy diet) and anti-health activities (e.g., cigarette smoking) [1].

Another definition says that health behaviours are all forms of behaviours people exhibit related to the health sphere, i.e., behaviours which, in light of modern medical knowledge, usually cause-specific (positive or negative) health effects [2] (pp. 23–57). They greatly affect the quality of life of individuals and are connected with one of the most important parts of human life, i.e., health. The patterns of health behaviour choices are governed by many factors (e.g., habits, the influence of family and peers, age, gender, place of residence, education level, socio-economic status, mental state, and psychological characteristics of an individual) [3] (pp. 46–58).

Among all the health behaviours an individual can undertake to try and increase their health potential, the literature on the subject distinguishes four groups [4]:

(1) Behaviour mainly related to physical health: taking care of the body and its immediate surroundings, physical activity, rational nutrition, or appropriate sleep duration and quality.
(2) Behaviour mainly related to psychosocial health: using and providing social support, avoiding excessive stress, or dealing with problems and tensions.
(3) Preventive behaviour: self-control of health, self-examination, taking preventive tests, safe behaviour in everyday life, or safe sex.
(4) Avoiding risky behaviours: not smoking, limited use of alcohol, not abusing non-prescribed medicines, or not using other psychoactive substances.

According to the World Health Organization (WHO, Geneva, Switzerland) perspective, health is considered a resource in everyday living, not simply the absence of disease. Thus, research into younger people’s health needs to consider the positive aspects of health, as well as risk factors for future ill health and disease. Many behaviours that comprise young people’s lifestyles may directly or indirectly impact their health in the short and long term; consequently, a wide range of behavioural variables should be measured. Positive or health-promoting behaviours need to be studied, as well as health-damaging or risky behaviour [5] (p. 8).

The adolescent and youth period are very important for adopting any health-related behaviors because they will continue throughout adulthood. Many health problems and disabilities in adulthood can be avoided if their related health risk behaviors are identified and changed at an early stage of life. Because it is also very challenging for adults to change unhealthy behaviors, it is vital to study lifestyle behaviors and their associated factors and then promote healthy lifestyle behaviors at younger ages.

The development of human beings passes through several transition phases throughout the life span. The most critical phase that may influence the individuals’ lifestyle is college admission. During this phase, the students are independent, and they are responsible for their own lives especially if they are far away from the parental home. A healthy lifestyle is identified by regular exercises, healthy diet, and organized sleeping pattern. However, the transfer into a new environment may alternate the usual habits and cause major fluctuations in lifestyle. The students may be vulnerable to several stressful factors including inability to organize time, stress of exams and deadlines, irregular sleeping patterns, new peer’s relationships, and inability to acclimatise to the new surroundings. These factors may result in a decreased level of physical activity and increased consumption of fast food that may lead to changes in body weight. The exposure to these changes in lifestyle may influence the well-being of an individual and overall health [6].

Many university students have more choices in health-related behaviors and are more prone to unhealthy lifestyles, shifting towards smoking, unhealthy nutrition, increased stress, and a sedentary lifestyle. They are formed as part of socialization and are influenced by a variety of personal factors and patterns in the home, peer group, local community, media, advertising, etc. ([7], pp. 1–7).

By starting their studies, young people increase their independence, forcing them to make decisions about their lifestyle, including those related to their health. Thus, they may be influenced by individual factors (e.g., taste preferences, self-discipline, and methods of spending time), social contacts (e.g., lack of parental control, and select friends and peers), selecting elements in their physical environment (e.g., availability, attractiveness, and prices of food products), and macroeconomic environment (e.g., media and advertising) ([8], pp. 3244–3255). This period of higher education for most students is the last stage before starting adult life. It is a time when preferences and needs in different spheres of life, including pro-health attitudes, are finally shaped.

University students’ lifestyle behaviors and health risk factors are important determinants of their ongoing health, as well as academic achievement and future career success. However, it is widely acknowledged, as a group, university students tend to have unhealthy lifestyle behaviors and high rates of health-related risk factor [9]. University is a period of responsibility in terms of choices and lifestyle practices, where students are exposed to the challenges of young adulthood while simultaneously tackling the mental and social issues of student life. University students represent the future of families, communities, and countries. As a consequence, they also face the stresses of achieving success in their academic goals while being expected to be competitive, adding to demands and burdens, which can lead to further stress. Many students confront changes in living conditions and health-promoting/damaging adjustments to lifestyle and environment [10].

College students are often busy with their studies and tend to spend a lot of time outside their homes and hence, may fall victim to unhealthy practices, gradually leading to an increase in body weight, high blood pressure, hypoglycemia and in effect to civilization diseases [11].

Cultural behaviors have important implications for human health. Culture, a socially transmitted system of shared knowledge, beliefs and/or practices that varies across groups, and individuals within those groups, has been a critical mode of adaptation throughout the history of our species [12].

The culture of a given society plays an important role in shaping all the behaviours related to health and disease. Culture is understood as a system of norms and beliefs, models of conduct, as well as all tangible and intangible products produced by a social group. Being born and raised in a single culture not only influences the person’s understanding of health and diseases but also influences how they react to them. In this context, health culture is a system of values attributed to physical and mental health, is subjective and objective, and is also personal and public. Health culture manifests itself in conscious regulation with human-environmental relations, and through that influencing the responsibility for one’s health, public health, and sensitivity to health needs. A certain health standard is achieved, one typical for a community, obtained thanks to the links between the culture of this community and its health [13].

A review of the literature on the topic in question shows that there are not many comparative studies of the lifestyle of students from four different countries. We want to compare and evaluate the lifestyles of students from multiple countries without division into years and fields of study. Thanks to this, the authors wanted to obtain universal knowledge, which would give the opportunity to undertake joint international attempts to improve the health condition of studying youth, and to facilitate their healthy activities. By paying attention to the multicultural aspect of the project, the results of the study could contribute to the fact that universities accepting foreign students will make every effort to ensure that studying, despite the fact that it is in a culturally different environment, facilitates taking actions promoting health.

This study aimed to compare and evaluate the lifestyles of students from selected countries in terms of preventive, risky, and health care behaviours, with regards to both physical and psychosocial health.

## 2. Material and Methods

The presented study was a part of a larger research project concerning pro-health and anti-health behaviour of students from selected countries. It was a cross-sectional, comparative study conducted from December 2016 to March 2017. The research was based on discussions with students in sociology and health promotion courses focused on their understanding of lifestyle in the context of maintaining and strengthening health. The major research question was: are there any differences in lifestyle between students from different countries? As a method was used a diagnostic survey and the survey technique.

### 2.1. Ethical Consideration

The study was conducted in accordance with the Declaration of Helsinki, and the protocol was approved by The Bioethics Committee of the Regional Chamber of Physicians and Dentists in Gdańsk No: KB-15/16.

### 2.2. Participants

The subjects were 532 students from Turkey, Poland, Greece, and Hungary. Respondents selected to participate in the survey were students of all fields of study. The authors selected students from partner countries with whom they have an agreement under European programs (Erasmus +). The first two authors of the study recruited at least two volunteers from each of the participating countries. These were students who had taken part in Erasmus+ activities in previous years whose role was to translate a research tool and a letter of intent into their national language and to send out a call for participants. The translated content was additionally verified for linguistic correctness by professional translators. This ensured the quality and linguistic accuracy of the translation. The questionnaire was adapted for comparative research utilizing cultural adaptation.

### 2.3. Tool

The original research tool included 85 closed questions. The selection of questions resulted from the literature on the subject, in which four groups of health-related behaviors were distinguished: on physical and psychosocial health-related behaviour, on preventive and risky behaviour, as well as questions to determine the socio-cultural profile of respondents. Due to the size of the study and the complexity of the data the authors chosen: eight questions concerning preventive behaviours, three—risky behaviours, 33—physical health, and five—psychosocial health. The level of acceptance of the student views was measured with a five-point Likert scale with a neutral choice: I strongly disagree, I disagree, I neither agree nor disagree, I agree, I strongly agree. The validation of the questionnaire fulfilled the requirements for translation and verification of the correctness of the questionnaire design [14].

### 2.4. Course of the Study

Recruitment was consecutive and snowball sampling was used. The non-random sampling method relies on the recruitment of participants by other participants. It is recommended as a sample selection method in order to facilitate scientific research and reaching the local community [15]. Each researcher distributed the survey questionnaire to their network of students, and they gave it their colleagues. Thanks to this, it was possible to reach the academic community in a given country.

The invitation letter informed potential participants of the aim of the survey, stated the name of the ethics committee/s which provided ethical approval for the study, and emphasized that participation was anonymous, confidential, and voluntary. Web-based electronic survey software was used to collect data in each country. The respondents were informed that their participation in the study was voluntary and that they would not be obligated to provide answers to any question(s) with which they were uncomfortable. The respondents were also informed that they could opt-out of the study at any time without any consequences. The subjects gave their informed consent for inclusion before they participated in the study.

A questionnaire was sent to students via the Internet. Participants were emailed a link and completed the survey at home or in their place of work. A paper questionnaire was provided for those participants who could not access the online survey. After the respondents’ answers were received, they were entered anonymously into the data collection worksheet. After data collection, each questionnaire was checked visually for completeness.

### 2.5. Methods of Statistical Analysis

Data were entered, cleaned, and coded using Statistica 8.0 PL software (StatSoft, Tulsa, OK, USA). The results were presented as a frequency table and descriptive statistics. The Kruskal-Wallis test (H) was used to verify the occurrence of differences between the groups. The most common answer (dominant) was defined, along with the frequency distribution for each variable, with an assumed level of confidence of *p* = 0.05.

## 3. Results

The study involved 532 students from the following fields of study: nursing, pedagogy, law, economics, national security, and geography. The following countries were selected: Greece, Poland, Turkey, and Hungary. Women accounted for 55.34% of the respondents, men 44.66%. The average weight was 63.2 kg, height—167.9 cm, and BMI—22.32 kg (Table 1). Of the respondents, 14.7% were treated for chronic diseases such as allergies, depression, diabetes, epilepsy, and thyroid diseases.

Of those surveyed 61.3% indicated they engaged in activities related to maintaining and improving health. They were most frequently undertaken in the area of psychosocial health—72.2% (Table 2) and avoiding risky behaviours—71.8% (Table 3), less frequently in the area of preventive behaviours—55.5% (Table 4), and physical health—45.6% (Table 5).

**Figure 1:**
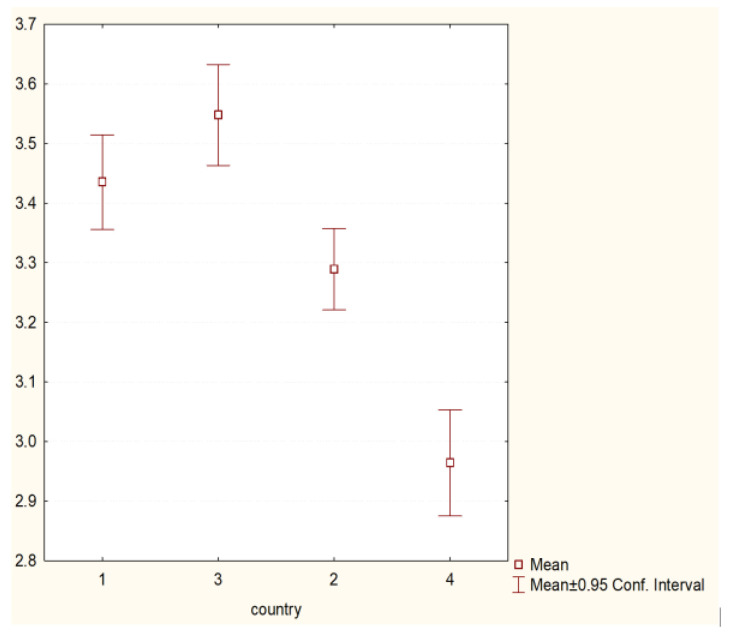
Preventive behaviours and country of origin (H = 98.43755 *p* = 0.000). Legend: 1—Poland, 2—Hungary, 3—Greece, 4—Turkey.

**Figure 2:**
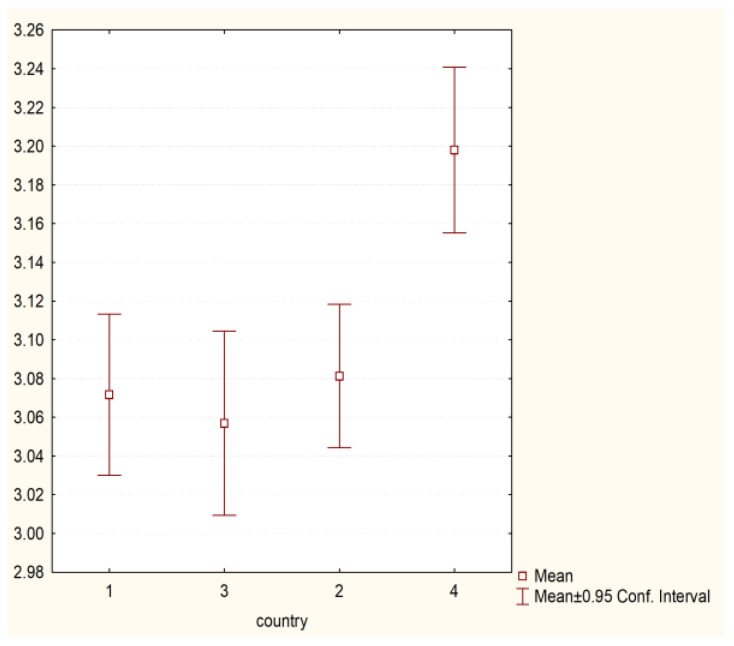
Physical health behaviours and country of origin (H = 22.70252 *p* = 0.0000). Legend: 1—Poland, 2—Hungary, 3—Greece, 4—Turkey.

**Figure 3:**
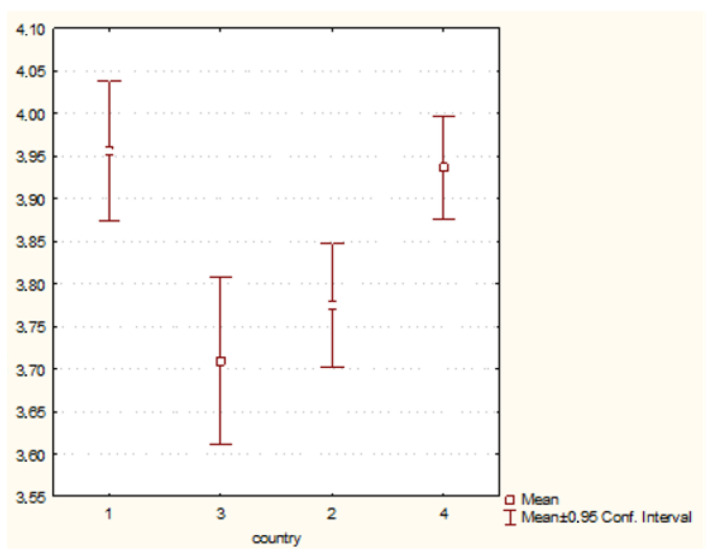
Psychosocial health behaviour and country of origin (H = 22.70252 *p* = 0.0000). Legend: 1—Poland, 2—Hungary, 3—Greece, 4—Turkey.

The analysis showed the existence of statistically significant differences between the behaviours undertaken by students from different countries presented within the framework:

(1) Preventive behaviour (H = 98.43755 *p* = 0.000)—Figure 1.
(2) Behaviour related to physical health (H = 22.70252 *p* = 0.0000)—Figure 2.
(3) Psychosocial health behaviour (H = 43.26644 *p* = 0.0000)—Figure 3.

**Figure 4:**
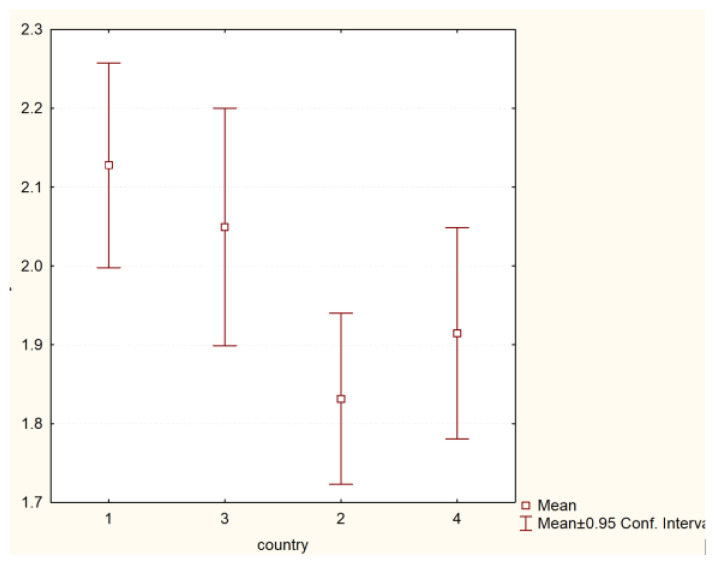
Risky behaviourso of students and the country of origin (H = 11.30513 *p* = 0.0102). Legend: 1—Poland, 2—Hungary, 3—Greece, 4—Turkey.

No statistically significant differences were found in the scope of students not taking risky behaviours (H = 11.30513 *p* = 0.0102)—Figure 4.

The highest psychosocial support was given to students from Poland (81.3%) and the lowest to students from Greece (17.4%). Most often, Hungarian respondents gave up undertaking risky behaviours (76.1%) and the least often were respondents from Poland (27.1%). Preventive behaviour was most often taken by the Greeks (60.5%) and least often by the Turks (30.4%). Physical health behaviours were most often preferred by students from Turkey (49.7%) and least frequently by Greeks (37.9%).

## 4. Discussion

The mobility and the possibilities of studying in places other than the country of origin, a broader view of students and their attitudes towards health, beyond the borders of the country in which they currently reside, is justified. The student population was selected as a result of their openness and susceptibility to change, as well as for the specificity of the health behaviours associated with higher education. However, the time of studying at university has many situations which force young people to behave in certain ways which can be temporary or possibly transferred to later in life ([16], pp. 89–93). Health behaviours are influenced not only by age and place in society, but also by socio-cultural environments. The correlations between health behaviours and culture are visible, among other factors including opinions on health risks, health evaluation, and observance of medical recommendations ([17,18], pp. 58–61).

The countries from which participants are from has different contributing factors such as standard of living, geographical origin, health care system, diet, religion, and so on. Despite the socio-cultural diversity of the respondents, their health-oriented lifestyle was similar. According to different authors, university students around the world in general do not have good eating habits, eating unbalanced diets high in calories, the physical exercise is nil, the high consumption of alcohol, tobacco and marijuana [19,20,21,22]. However, in some areas of health, there are differences. They are at the same time a result of individual behaviour and social and cultural influence.

In the discussion of the obtained results, due to the multiplicity of contexts, attention was paid to those that could have had an impact on the results during the research. It should also be taken into account that the quoted and compared data are adequate to the duration of the research.

Psychosocial health was most often taken care of by respondents from Poland. A possible basis for such behaviour could be a result of Polish society’s family-oriented culture. The results of opinion polls show Polish people often spend their free time primarily with their family and among friends. Family, family happiness (82%), and health (74%) are highly valued [23]. An independent social determinant of health is the extent, strength, and quality of our social connections with others. The recognition of the importance of social connections for health dates back to as far as the work of Emil Durkheim. Bowlby [24] maintained that secure attachments are not only necessary for food, warmth, and other material resources, but also provide love, security, and other non-material resources that are necessary for normal human development [25].

Certain periods in life may be critical for the development of bonds and attachments ([26], pp. 125–151). The provision and reception of social support is an important element in health and is part of the basis for meeting the basic needs of love, belonging, and acceptance. Whether one is facing a personal crisis and needs immediate help, or simply wants to spend time with people who care for them, these relationships play a key role in everyday life.

Alcohol consumption, smoking, and the use of psychoactive and addictive substances are behaviours that cause undesirable health effects, increasing the risk of developing lifestyle diseases and increasing mortality rates. Therefore, in most countries around the world, NGOs and various organisations run multifunctional campaigns aimed at reducing the incidence of adverse events and thus increase awareness of the negative effects of substance abuse [27].

In this study, risky behaviours were least frequently undertaken by Hungarians. According to WHO data, the awareness of the Hungarian population has changed in recent years, which is noticeable, for example, in the amount of alcohol consumed. In the years 2003–2005 it was 17.1 L per person, in the years 2008–2010 it was 13.3 L and in 2014 it was 10.9 L per adult. WHO data from 31 December 2016 also show that in Hungary 25.8% of the population smoked cigarettes, which is a less common than in the other examined countries, e.g., Greece, where 27.3% of the adult population smoke [28]. Positive trends in avoiding risky behaviours are also confirmed by data from research carried out in March 2015 by the Hungarian Scientific Research. However, compared to findings of former ESPAD surveys it can be stated that by 2015 the prevalence of all illicit substances has decreased markedly and substance use has become rarer among Hungarian high school students [29].

A growing number of multilevel studies have found an association between community stocks of social capital and individual health outcomes (e.g., mortality, self-rated health, some health behaviours) regardless of the influence of individual socioeconomic characteristics ([30], pp. 682–690).

The correlation between health behaviours and standard of living in a country is an issue greatly discussed in the literature [31,32,33].

The economic-debt crisis that hit Greece form early 2009 to late 2018 has affected all aspects of society, including the state of health, which the EU considers to be considerably low here ([33], pp. 597–602). Both access to health services and the quality of health services can impact health. Lack of access or limited access to health services greatly impacts an individual’s health status. Given the level of quality of Greek healthcare, society was forced to move towards more self-care measures. It, therefore, cannot be ruled out that the country’s economic situation resulted in Greek students taking greater care of their health than students from other countries, enforcing self-control and self-examination, taking preventive examinations and practising safe behaviours in everyday life.

Turkish people seem to care about physical health, i.e., body hygiene, diet, and sleep, and it is possibly closely connected with their culture. Turkish culture may be characterized as having elements of modernity, tradition, and Islamic practices ([34], pp. 833–874); cultural orientations are not homogenous and are not equally adopted by citizens. Modern-day Turkey is a secular state: however, the philosophy and ideology of Islam remains a prevalent feature of Turkish culture. In Turkish culture, care for the body, clothing, and the immediate environment is documented in the Quran [35]. Important everyday activities include hand washing, taking care of one’s nails, hair, sexual organs, clothing, and diet. For this reason, among others, the concerns for the body is strongly inscribed in the daily behaviours of Turkish people. This seems to be undoubtedly a strong factor favouring positive physical health behaviours undertaken by Turkish students, more so than by other respondents.

Universities may be excellent settings to address misperceptions and to influence norms regarding body weight in order to prevent unhealthy behaviours among students to achieve ill-advised weight ideals [36].

The BMI of the students participating in the study was within the norms according to WHO [37], but considering the eating habits of students and the fact that 32% attach importance to the diet, it should be recognised, that the time of study may be the time when pro-health or anti-health behaviors become established, leading to diseases in the future.

Another important issue raised by other authors is the regularity of meals, which is problematic among students [38,39].

For the respondents of the survey in question, 62.1% of physical activity is important in life, but 41.7% spend their free time actively and 31.7% exercise regularly at least 3 times a week. The disproportion between the declaration and the activity undertaken is significant.

Researchers who reviewed studies on college students’ physical activity behaviors reported that about 40% to 50% of college students are physically inactive [40].

The new Global Action Plan on Physical Activity 2018–2030 of the WHO promotes sporting and physical activity as a leading factor for mental health, quality of life and wellness [41].

Students who took part in the study often acknowledged health oriented activities such as taking care of the body and immediate surroundings, physical activity, well-balanced nutrition, taking care of sleep, using social support, coping with problems and stress, controlling health, taking preventive examinations, not smoking, limited alcohol consumption, not using other psychoactive substances, however, there was still a large group of people who did not follow the recommendations to promote a healthy lifestyle.

The unhealthy behaviours reported in this study identify these students as having a high risk for future chronic diseases and premature mortality [42,43]. The presented results do not differ from those presented by other authors conducting research on students [44,45] (pp. 1910–1919), ([46], pp. 315–318), ([47], p. 97–104), ([48], pp. 171–179). The findings reflect international research on hazardous drinking, cigarette smoking, unhealthy diet/eating patterns, low levels of physical activity, and unsafe sex among student populations.

Higher education is becoming one of the main factors for a successful career in the labour market and a better perspective on living conditions. Through their example and decisions, intelligent health professionals with the skills to take independent action to maintain and improve their health will be able to promote good behaviour to their loved ones and peers in the workplace ([49], pp. 289–303).

Nowadays, there is a strong emphasis on health promotion as a science and it can help people transform their lifestyles and optimize their health. In this context, it is worth making young people aware of their potential for better health now and in the future. It is up to them to take care of their health now to ensure that they increase their quality of life in their later years. It is important to stress that health is not a fixed value. It is subject to fluctuations and it will depend on them whether it will trend downward or upward in the future. Young people should heed the motto “My health in my hands”.

## 5. Limitations

The conducted research, although it provides important knowledge, is subject to several limitations. Conducting research via the Internet seems to be a great help in the case of intercultural research. Being able to access respondents from different cultures in their own environment is easier, simpler, faster and cheaper. However, there are many difficulties in conducting research using the Internet. They concern both technical and practical as well as content-related and methodological issues. The first of the difficulties of research via the Internet is the recruitment of people, which, as in this study, is carried out in a non-random manner, using the snowball method (the so-called chain), where the research involves people who know each other and who recommend participation in the research to other people they know. Thanks to this, it is possible to reach a specific group of people, here students, but also a group of friends who probably show a similar style and philosophy of life, which reduces the diversity of the group.

Moreover, in our study the nationality of the respondents was taken into account as an independent variable. However, the aforementioned openness of borders, the ability to move freely, use various discussion forums or social media means that people’s habits are subject to change, and as a result they cease to be specific and characteristic for a given geographical area, nation or culture.

## 6. Conclusions

All surveyed students declare that they undertake health-promoting activities, however, there are significant differences with regard to health behaviors between students depending on the country of origin. Students from Poland received the greatest support with regards to psychosocial health and most often exhibited risky behaviours. Students from Hungary had the highest BMI index and most often did not show risky behaviours. Students from Greece received the least support with regards to psychosocial health, most commonly showed preventive behaviour, and least frequently engaged in physical health behaviours. Students from Turkey had the lowest BMI index, most often engaged in physical health behaviours, and least often exhibited preventive behaviour. Students should be supported in their self-care by ensuring they are well educated and able to acquire new skills for health promotion.

Our conclusions could be guidelines for university authorities to make them a place that promotes a healthy lifestyle, facilitate maintaining health and teach how to care for it. Identifying areas in which students show deficiencies in the care of health could be a premise for introducing changes in the functioning of the university, e.g., changing the menu in university canteens, facilitating access for students to university gyms, sports clubs, or employing a psychologist, etc.

Meetings of international project coordinators, such as Erasmus is also a good place to discuss health in a cultural context. Research shows that planned health education among students, promoting a healthy lifestyle in various dimensions, improves pro-health attitudes [50].

## Figures and Tables

**Table 1:** Characteristics of the studied population.

Parameter	Poland	Hungary	Greece	Turkey
	*n* = 154	*n* = 170	*n* = 95	*n* = 113
Gender				
Women	74 (48.1%)	83 (48.8%)	50 (52.6%)	54 (47.8%)
Men	80 (51.9%)	87 (51.2%)	45 (47.4%)	59 (52.2%)
Mean weight (kg)	62.5	67.2	68.0	54.9
Mean height (cm)	168.0	166.7	171.6	165.4
Mean BMI	22.32	24.02	22.99	19.96

**Table 2:** Behaviour mainly related to psychosocial health: using and providing social support, avoiding excessive stress and dealing with problems and tensions.

No.	Statements	I Strongly Disagree (%)	I Disagree (%)	I Neither Agree Nor Disagree (%)	I Agree (%)	I Strongly Agree (%)
1	I have someone who is with me during hard situations	3.1	6.6	18.6	39.0	32.7
2	I have someone who tries to engage me in activities that help me forget about my problems	3.4	4.8	9.3	40.0	42.5
3	I feel supported when it comes to my goals and plans for achieving them	3.1	6.9	15.9	54.0	20.1
4	In difficult situations I can count on my family’s or partner’s support	3.4	5.2	7.9	36.4	47.1
5	In difficult situations I can count on my friends’ help	3.8	3.8	15.9	45.0	31.5

**Table 3:** Avoiding risky behaviours: not smoking, limited use of alcohol, not abusing medicines not prescribed by a doctor, not using other psychoactive substances.

No.	Statements	I Strongly Disagree (%)	I Disagree (%)	I Neither Agree Nor Disagree	I Agree (%)	I Strongly Agree (%)
1	I do not smoke cigarettes	57.0	13.8	5.5	12.8	10.0
2	I do not drink alcohol	33.7	22.0	16.2	21.3	6.8
3	I do not use drugs or other substances	74.1	12.8	6.2	5.2	1.7

**Table 4:** Preventive behaviour: self-control of health and self-examination, taking preventive tests, safe behaviour in everyday life, safe sex.

No.	Statements	I Strongly Disagree (%)	I Disagree (%)	I Neither Agree Nor Disagree (%)	I Agree (%)	I Strongly Agree (%)
1	I pay attention to my health	1.7	7.5	15.6	52.6	22.7
2	I control the state of my health by regular check-ups	4.7	17.3	24.7	43.1	10.2
3	I follow the doctor’s instructions when I’m ill	1.4	4.9	12.2	53.1	28.4
4	I go to the doctor when I feel ill	2.4	17.7	22.6	42.0	15.3
5	I treat myself when I feel ill	3.4	14.5	23.8	43.4	14.9
6	I practice safe sex	12.1	10.7	11.4	29.3	36.5
7	I change my sexual partners rarely	2.4	4.5	6.6	19.0	67.5
8	I use contraception	25.5	16.4	13.6	18.9	25.6

**Table 5:** Behaviour mainly related to physical health: taking care of the body and its immediate surroundings, physical activity, rational nutrition, sleep of an appropriate duration and quality.

No.	Statements	I Strongly Disagree (%)	I Disagree (%)	I Neither Agree Nor Disagree (%)	I Agree (%)	I Strongly Agree (%)
1	Diet plays an important role in my life	14.3	28.6	25.1	21.3	10.7
2	I prepare my own meals	2.4	8.9	19.9	50.9	35.9
3	I eat regularly every day	9.0	16.2	24.8	37.9	12.1
4	I eat fruits every day	5.9	17.0	19.0	44.6	13.5
5	I eat vegetables every day	5.2	20.7	19.7	39.7	14.7
6	I eat regularly every day	4.5	17.9	27.6	38.6	11.4
7	I eat fruits every day	9.0	30.7	22.4	30.3	7.6
8	I eat vegetables every day	10.1	37.0	18.4	25.9	8.6
9	I do not eat products after their expiration date	3.8	9.3	13.4	25.2	48.3
10	Physical activity is an important part of my life	5.2	11.0	21.7	46.9	15.2
11	I spend my free time on some physical activity	11.1	19.8	27.4	34.7	7.0
12	I participate in selected physical activity 3 times a week or more often	13.1	30.0	25.2	22.4	9.3
13	I spent more than 30 min every time on physical activity	10.8	20.1	20.1	31.6	17.4
25	I sleep 7-8 h every night	8.2	19.9	15.1	36.4	20.4
26	I miss sleep because of studying	13.7	29.0	25.2	19.0	13.1
27	I miss my sleep because I’m partying	7.3	11.8	18.4	29.5	33.0
28	I wake up rested	6.6	21.6	30.0	28.2	13.6
29	I bathe every day or shower my entire body	1.7	8.9	9.3	24.4	55.7
30	I wash my teeth at least twice every day	1.7	10.3	8.2	30.1	49.1
31	I wash my hands during the day	2.1	1.0	3.8	21.6	71.5
32	I keep my room neat and tidy	2.8	5.5	13.4	39.0	39.3
33	I build up my resistance	1.7	7.2	20.6	45.4	25.1

## Data Availability

The data analyzed in the study are available upon request to the first author.

## References

[1] Steptoe A., Gardner B., Wardel J. (2010). The role of behaviour in health. Healh Psychology.

[2] Short S.E., Mollborn S. (2015). Social determinants and health behaviors: Conceptual frames and empirical advances. Curr. Opin. Psychol..

[3] Kluczyńska U., Cylkowska-Nowak M. (2008). Styl życia. Główne Podejścia i Perspektywy Badawcze. Edukacja Zdrowotna. Możliwości, Problemy, Ograniczenia.

[4] Woynarowska B., Woynarowska B. (2008). Edukacja zdrowotna—podstawy teoretyczne i metodyczne. Edukacja Zdrowia.

[5] Currie C., Currie C., Hurrelmann K., Settertobulte W., Smith R., Todd J. (2000). The international HBSC Study: Rationale, history and description. Health and Health Behaviour among Young People. Health Behaviour in School-aged Children: A WHO Cross-National Study (HBSC) International Report.

[6] Assaf I., Brieteh F., Tfaily M., El-Baida M., Kadry S., Balusamy B. (2019). Students university healthy lifestyle practice: Quantitative analysis. Health Inf. Sci. Syst..

[7] Alzahrani S.H., Malik A.A., Bashawri J., Shaheen S.A., Shaheen M.M., Alsaib A.A., Mubarak M.A., Adam Y.S., Abdulwassi H.K. (2019). Health-promoting lifestyle profile and associated factors among medical students in a Saudi University. SAGE Open Med..

[8] Eyre H., Kahn R., Robertson R.M. (2004). Preventing cancer, cardiovascular disease, and diabetes: A common agenda for the American Cancer Society, the American Diabetes Association, and the American Heart Association. Circulation.

[9] Whatnall M.C., Patterson A.J., Brookman S., Convery S., Swan C., Pease S., Hutchesson M.J. (2019). Lifestyle behaviors and related health risk factors in a sample of Australian university students. J. Am. Coll. Health.

[10] Nasir U., Butt A.F., Choudry S. (2019). A Study to Evaluate the Lifestyle of Medical Students in Lahore, Pakistan. Cureus.

[11] Syed N.K., Syed M.H., Meraya A.M., Albarraq A.A., Al-Kasim M.A., Alqahtani S., Makeen H.A., Yasmeen A., Banji O.J.F., Elnaem M.H. (2020). The association of dietary behaviors and practices with overweight and obesity parameters among Saudi university students. PLoS ONE.

[12] Hernandez M., Gibb J.K. (2020). Culture, Behavior and Health. Evol. Med. Public Health.

[13] Papazoglou G. (2019). Society and Culture: Cultural Policies Driven by Local Authorities as A Factor in Local Development—The Example of the Municipality of Xanthi-Greece. Heritage.

[14] Tafforeau J., Cobo M.L., Tolonen H., Scheidt-Nave C., Tinto A. Guidelines for the Development and Criteria for the Adoption of Health Survey Instruments. https://ec.europa.eu/health/ph_information/dissemination/reporting/healthsurveys_en.pdf.

[15] Ghaljaie F., Naderifar M., Goli H. (2017). Snowball sampling: A purposeful method of sampling in qualitative research. Strides Dev. Med. Educ..

[16] Romanowska-Tołłoczko A. (2011). Styl życia studentów oceniany w kontekście zachowań zdrowotnych. Hygeia Publ. Health.

[17] Matsumoto D., Juang L. (2007). Psychologia Międzykulturowa.

[18] Wijata J., Jędrzejczak M. (2007). Socjomedyczne determinanty kultury zdrowotnej pacjentów lekarza pierwszego kontaktu—Próba pomiaru. Med. Rodz..

[19] Fabián C., Pagán I., Ríos J.L., Betancourt J., Cruz S.Y., González A.M., Palacios C., Gonzales M.J., Rivera-Soto W.T. (2013). Dietary Patterns and their Association with Socio- demographic Characteristics and Perceived Academic Stress of College Students in Puerto Rico. Health Sci. J..

[20] Martinez-Lacoba R., Pardo-Garcia I., Amo-Saus E., Escribano-Sotos F. (2018). Socioeconomic, demographic and lifestyle-related factors associated with unhealthy diet: A cross-sectional study of university students. BMC Public Health.

[21] Smith L., Disler R., Watson K. (2020). Physical activity and dietary habits of first year nursing students: An Australian dual-method study. Collegian.

[22] Alhazmi A., Aziz F. (2020). Dietary assessment and its awareness in female students from different Health Departments: Unhealthy diet with normal BMI. J. Public Health Res..

[23] CBOS, Komunikat z Badań Wartości i Normy. Warszawa 2013. https://www.cbos.pl/SPISKOM.POL/2013/K_111_13.PDF.

[24] Bowlby J. (1982). Attachment and Loss. Vol. 1. Attachment.

[25] Berkman L., Glass T., Berkman L., Kawachi I. (2000). Social integration, social networks, social support, and health. Social Epidemiology.

[26] Winston R., Chicot R. (2016). The importance of early bonding on the long-term mental health and resilience of children. London J. Prim. Care.

[27] National Institute for Health and Welfare, The Action Plan on Alcohol, Tobacco, Drugs and Gambling. https://thl.fi/fi/web/thlfi-en/research-and-expertwork/projects-and-programmes/the-action-plan-on-alcohol-tobacco-drugs-and-gambling,on-line:08.08.2018.

[28] OECD and World Health Organization State of Health in the EU Hungary Country Health Profile 2017. https://www.euro.who.int/__data/assets/pdf_file/0006/355983/Health-Profile-Hungary-Eng.pdf.

[29] ESPAD 2020 Report Results from the European School Survey Project on Alcohol and Other Drugs. https://www.emcdda.europa.eu/system/files/publications/13398/2020.3878_EN_04.pdf.

[30] Ehsan A., Klaas H.S., Bastianen A., Spini D. (2019). Social capital and health: A systematic review of systematic reviews. SSM Popul. Health.

[31] Molarius A., Berglund K., Eriksson C., Lambe M., Nordström E., Eriksson H.G., Feldman I. (2007). Socieconomic conditions, lifestyle factors, and self-rated health among men and women in Sweden. Eur. J. Public Health.

[32] Goryakin Y., Lobstein T., James T., Suhrcke M. (2015). The impact of economic, political and social globalization on overweight and obesity in the 56 lowand middle income countries. Social Sci. Med..

[33] Niakas D. (2013). Greek Economic Crisis and Health Care Reforms: Correcting the Wrong Prescription. Int. J. Health Serv..

[34] Kabasakal H., Bodur M., Chhokar J.S., Brodbeck F.C., House R.J. (2007). Leadership and Culture in Turkey: A Multi-faceted Phenomenon. Culture and Leadership Across the World the GLOBE Book of In-Depth Studies of 25 Societies.

[35] Quran Surah Al-A’raf, Ayat 7: 31, Czachorowski, M.C. (transl.), Muzułmański Związek Religijny w RP Najwyższe Kolegium Muzułmańskie 1439/2018 (in Polish). http://bibliotekamuzulmanska.pl/wp-content/uploads/2018/10/koran.pdf.

[36] Mikolajczyk R.T., Maxwell A.E., El Ansari W., Stock C., Petkeviciene J., Guillen-Grima F. (2010). Relationship between perceived body weight and body mass index based on self-reported height and weight among university students: A cross-sectional study in seven European countries. BMC Public Health.

[37] Nuttall F.Q. (2015). Body mass index: Obesity, BMI, and health: A critical review. Nutr. Today.

[38] Scott-Sheldon L.A., Carey K.B., Carey M.P. (2008). Health behavior and college students: Does Greek affiliation matter?. J. Behav. Med..

[39] Farshchi H.R., Taylor M.A., MacDonald I.A. (2005). Beneficial metabolic effects of regular meal frequency on dietary thermo- genesis, insulin sensitivity, and fasting lipid profiles in healthy obese women. Am. J. Clin. Nutr..

[40] Keating X.D., Guan J., Piñero J.C., Bridges D.M. (2005). A meta-analysis of college students’ physical activity behaviors. J. Am. Coll. Health.

[41] D’Anna C., Forte P., Gomez F. (2019). Physical education status in European school’s curriculum, extension of educational offer and planning. J. Hum. Sport Exer..

[42] (2001). Health and Behavior: The Interplay of Biological, Behavioral, and Societal Influences.

[43] Suhrcke M., Nugent R.A., Stuckler D., Rocco L. (2006). Chronic Disease: An Economic Perspective.

[44] Deliens T., Clarys P., de Bourdeaudhuij I., Deforche B. (2014). Determinants of eating behaviour in university students: A qualitative study using focus group discussions. BMC Public Health.

[45] Sánchez-Ojeda M.A., De Luna-Bertos E. (2015). Healthy lifestyles of the university population. Nutr. Hosp..

[46] Tamanal J.M., Kim C.H. (2020). Promoting Healthy Lifestyle in High School Students: Determination of the Lifestyle Status through the Healthy Lifestyle Screen (HLS) Assessment. J. Lifestile Med..

[47] Melnyk B.M., Jacobson D., Kelly S., Belyea M., Shaibi G., Small L., O’Haver J., Marsiglia F.F. (2013). Promoting healthy lifestyles in high school adolescents: A randomized controlled trial. Am. J. Prev. Med..

[48] Uramowska-Żyto B., Kozłowska-Wojciechowska A., Jarosz M., Makarewicz-Wujec M. (2004). Wybrane elementy stylu życia studentów wyższych uczelni w świetle badań empirycznych. Roczn PZH.

[49] Lisicki T. (2014). Zainteresowanie studentów Uniwersytetu Zielonogorskiego uzyskiwaniem wiedzy o zdrowiu. Roczn Lub..

[50] Solhi M., Azar F.E.F., Abolghasemi J., Maheri M., Irandoost S.F., Khalili S. (2020). The effect of educational intervention on health-promoting lifestyle: Intervention mapping approach. J. Edu. Health Prom..

